# Atypical Complications of Graves' Disease: A Case Report and Literature Review

**DOI:** 10.1155/2017/6087135

**Published:** 2017-02-28

**Authors:** Khaled Ahmed Baagar, Mashhood Ahmed Siddique, Shaimaa Ahmed Arroub, Ahmed Hamdi Ebrahim, Amin Ahmed Jayyousi

**Affiliations:** ^1^Endocrine Department, Hamad Medical Corporation, Doha, P.O. Box 3050, Qatar; ^2^Emergency Department, Hamad Medical Corporation, Doha, P.O. Box 3050, Qatar

## Abstract

Graves' disease (GD) may display uncommon manifestations. We report a patient with rare complications of GD and present a comprehensive literature review. A 35-year-old woman presented with a two-week history of dyspnea, palpitations, and edema. She had a raised jugular venous pressure, goiter, and exophthalmos. Laboratory tests showed pancytopenia, a raised alkaline phosphatase level, hyperbilirubinemia (mainly direct bilirubin), and hyperthyroidism [TSH: <0.01 mIU/L (reference values: 0.45–4.5), fT4: 54.69 pmol/L (reference values: 9.0–20.0), and fT3: >46.08 pmol/L (reference values: 2.6–5.7)]. Her thyroid uptake scan indicated GD. Echocardiography showed a high right ventricular systolic pressure: 60.16 mmHg. Lugol's iodine, propranolol, cholestyramine, and dexamethasone were initiated. Hematologic investigations uncovered no reason for the pancytopenia; therefore, carbimazole was started. Workup for hepatic impairment and pulmonary hypertension (PH) was negative. The patient became euthyroid after 3 months. Leukocyte and platelet counts and bilirubin levels normalized, and her hemoglobin and alkaline phosphatase levels and right ventricular systolic pressure (52.64 mmHg) improved. This is the first reported single case of GD with the following three rare manifestations: pancytopenia, cholestatic liver injury, and PH with right-sided heart failure. With antithyroid drugs treatment, pancytopenia should resolve with euthyroidism, but PH and liver injury may take several months to resolve.

## 1. Introduction

Graves' disease (GD) is an autoimmune thyroid disease first described by Robert Graves in 1835 [1]. Patients with GD usually present with common manifestations such as palpitations, tremors, heat intolerance, and weight loss. However, some patients may present with unusual gastrointestinal, hematologic, neurologic, and cardiopulmonary complications [2]. We report a patient who presented to our institution with pancytopenia and pulmonary hypertension (PH) with right-sided heart failure and raised liver enzymes. She was found to have GD. Further, there was no other explanation for her manifestations as the workup failed to reveal any other pathologic conditions. The pathogenesis of these complications in patients with GD is not completely understood. However, the prognosis is good if the underlying hyperthyroidism is treated with antithyroid drugs (ATDs). In this review, we discuss the prevalence of each complication, possible mechanisms of their development in the context of GD, and the clinical course during treatment of the underlying hyperthyroidism.

## 2. Case Presentation

A 35-year-old woman presented to our emergency department with a two-week history of breathlessness, palpitations, and generalized edema. She had a history of having lost approximately 10 kg of weight over a 3-month period but had regained weight in the month before presentation. She had no chronic illness and was not on any medication(s). On examination, she looked tired, was afebrile, and had a blood pressure of 103/58 mmHg, pulse rate of 92/min, and a respiratory rate of 20/min. She had exophthalmos, a raised jugular venous pressure, a diffuse goiter with a positive bruit, and bilateral pedal edema extending to the thighs. She had a third heart sound and bilateral fine basal lung crepitations. Her abdominal examination was unremarkable with no tenderness or organomegaly. An electrocardiogram showed a sinus rhythm with right bundle branch block and right ventricular strain.

Laboratory investigations (detailed in Table 1) showed pancytopenia; raised bilirubin (mainly direct bilirubin) and alkaline phosphatase (ALP); and normal creatinine, alanine aminotransferase (ALT), and aspartate aminotransferase (AST) levels. Her thyroid functions were as follows: thyroid-stimulating hormone (TSH), <0.01 mIU/L (reference values: 0.45–4.5); FT4, 54.69 pmol/L (reference values: 9–20); FT3, >46.08 pmol/L (reference values: 2.6–5.7); and antithyroid peroxidase, >1000 U.

Echocardiography showed an ejection fraction of 50–55%, a right ventricular systolic pressure (RVSP) of 60.16 mmHg measured by Doppler, and severe tricuspid regurgitation. Therefore, a computed tomography pulmonary angiogram was performed; it was negative for pulmonary embolism. A thyroid uptake scan showed diffusely increased uptake, indicating GD, and ultrasound examination showed a diffuse goiter with no nodules. She was initially started on Lugol's iodine, 7 drops (8 mg/drop) every 8 hours, propranolol, 40 mg every 8 hours, dexamethasone, 1 mg every 8 hours, and cholestyramine, 4 grams every 6 hours, to control thyrotoxicosis and to prevent further worsening of the patient's complications which were suspected to be related to GD. Also, 2 doses of intravenous furosemide 40 mg were given on the first hospital day then it was discontinued and the basal lungs crepitations disappeared on the following day.

Workup did not reveal any specific reason other than GD for her pancytopenia, PH, and raised alkaline phosphatase (ALP) and bilirubin. Regarding the pancytopenia, a peripheral blood smear showed a moderate normocytic anemia with mild hypochromia; a few ovalocytes, burr cells, and schistocytes; and increased rouleaux formation. There was leukopenia with neutropenia, some toxic features, a few reactive lymphocytes, and minor platelet clumps. The reticulocyte count was 3.2% (reference values: 0.5–2.5%), erythrocyte sedimentation rate was 3 mm/h (reference values: 2–37), C-reactive protein was 9 mg/L (reference values: 0–5), and hemoglobin electrophoresis was normal. The levels of all the following parameters were normal: lactate dehydrogenase, 184 U/L (reference values: 135–214); haptoglobin, 95 mg/dL (reference values: 35–250); folate, 34.5 nmol/L (reference values: 4.0–45.3); and vitamin B12, 617 pmol/L (reference values: 133–675). Iron studies showed the following: iron, 5 *μ*mol/L (reference values: 5.83–34.5); total iron binding capacity, 32 *μ*mol/L (reference values: 45–80); iron saturation, 15% (reference values: 15–45); transferrin, 1.29 g/L (reference values: 2–3.6); and ferritin, 38 *μ*g/L (reference values: 11–304). Coombs test and HIV serology were negative. Additional workup for cholestatic liver impairment, viral hepatitis serology, and antimitochondrial antibodies (AMA) was negative. The patient's abdominal ultrasound was unremarkable, with no hepatic or splenic enlargement. Further investigations for PH, rheumatoid factor, and antinuclear (ANA), anti-neutrophil cytoplasmic, anti-RO, anti-LA, anti-JO, anti-scleroderma 70, anti-RNP, and anti-smith antibodies were negative. Complements 3 (C3) and 4 (C4) were 110 mg/dL (reference values: 90–180) and 18.4 mg/dL (reference values: 10–40), respectively.

Bone marrow examination was not performed as the reticulocyte count was high and in the following two days of the initial treatment patient's white blood cells and platelets improved; at that point carbimazole 60 mg/day was started. After six days, she was ready for discharge from hospital on the same dose of carbimazole and propranolol, and furosemide was added for the lower limbs edema.

Follow-up investigations nine days later showed improvement in white blood cell and platelet counts. In addition, the gamma-glutamyl transferase (GGT) level was high (128 U/L [normal: 9–36]).

Two months after her initial presentation, the lower limb edema had improved but had not resolved completely. A month later (3 months after presentation), the lower limb edema disappeared. Moreover, the clinical and laboratory features of hyperthyroidism resolved with carbimazole therapy, and her bilirubin level and white blood cell and platelet counts normalized; and her hemoglobin, ALP, and GGT levels improved. ALP isoenzymes were checked; the raised ALP level was mainly of hepatic origin. Repeat echocardiography showed improvement of RVSP (52.64 mmHg) measured by Doppler, with moderate tricuspid regurgitation.

## 3. Discussion

GD is caused by autoantibodies that stimulate TSH receptors in the thyroid gland; it is the most common cause of hyperthyroidism [3]. Our patient is the first reported case to have these three rare complications of GD: pancytopenia, cholestatic hepatic injury, and PH with right-sided heart failure.

### 3.1. Pancytopenia

It is rare for patients with GD to have pancytopenia; there are only a few documented reports in the literature [1, 4–9]. However, single lineage abnormalities (anemia, leukopenia, or thrombocytopenia) are more common in patients with hyperthyroidism (34%, 5.8%, and 3.3%, resp.) [5, 10]. Leukopenia with a relative lymphocytosis is not an uncommon blood abnormality in GD, named “Kocher's blood picture.” [11]. There was no identifiable cause for this patient's anemia and it was similar to an anemia of chronic disease, commonly called “GD anemia,” that affects 22% of patients with GD [12]. Bone marrow examination in patients with GD, although was not performed in our case, could show hypercellular [8], normocellular [1, 5, 6], or, very rarely, hypoplastic [4] changes.

Initially, thionamides were not used. We chose a more conservative approach to treatment in this patient with multiple complications, using alternatives such as Lugol's solution and cholestyramine, as we did not want to aggravate her pancytopenia. The use of radioactive iodine at the time of presentation might have precipitated a thyroid storm [13]; thus, it was not considered. However, once the workup for pancytopenia showed no other causes and the neutrophil and platelet counts were improving, we initiated treatment with carbimazole. Generally, the use of thionamides is contraindicated in patients with a baseline neutrophil count <0.5 × 10^9^/L [13] and should be stopped if the neutrophil count drops after their initiation below 1.0 × 10^9^/L [14]. Thus, although ATDs can be used in patients with hyperthyroidism-associated pancytopenia, their use is recommended only after a full hematologic assessment has been performed, as ATDs carry a minimal risk of pancytopenia [15]. Dexamethasone was added to the initial management because of the severe thyrotoxic state of the patient with pancytopenia. However, the evidence for its use in this situation was weak, being derived from a previous case report [5].

The duration of hyperthyroidism related pancytopenia is variable, from 2 weeks [1] to several months [4]; it resolves only when the patient is euthyroid. Pancytopenia should resolve by the time euthyroidism is reestablished; if it does not, hematologic evaluation should be revisited. It was reported that a patient with GD-related pancytopenia started on methimazole showed no improvement despite becoming euthyroid; investigations revealed methimazole-associated aplastic anemia [8].

The pathogenesis of GD-related pancytopenia is not fully understood and different theories exist [1, 4, 5, 10], including the following: (i) high level of circulating thyroid hormones, resulting in ineffective hematopoiesis; (ii) shortened blood cell lifespan, either by immune destruction or by sequestration; (iii) autoimmune mechanisms, given the presence of antineutrophil and antiplatelet antibodies; and (iv) direct bone marrow toxicity, as excessive thyroid hormones may adversely affect the pluripotent stem cells.

The mechanism of pancytopenia is most likely multifactorial as no single theory could explain it completely. The association between GD and other autoimmune hematologic disorders that respond to ATDs (e.g., autoimmune hemolytic anemia, immune thrombocytopenic purpura, and Evan's syndrome [6]) supports the autoimmune theory. However, pancytopenia has also been reported with other forms of hyperthyroidism that are not immunologically mediated, such as toxic multinodular goiter [10], toxic adenoma [16], and even levothyroxine overtreatment in a patient previously treated for GD [17].

### 3.2. Cholestatic Hepatic Injury

The hepatic derangement in patients with GD ranges from mild laboratory abnormalities without clinical features to overt hepatitis [18], with either hepatitic or cholestatic injury [19]. Our patient had a cholestatic pattern of liver impairment with raised bilirubin (predominantly direct bilirubin), ALP, and GGT. After 3 months of carbimazole treatment with resolution of hyperthyroidism, ALP and GGT levels were better, and bilirubin normalized. Moreover, ANA and AMA were negative, thereby excluding concomitant autoimmune liver disease such as primary biliary cirrhosis. The patient had no clinical or ultrasonographic manifestations of hepatic congestion (i.e., hepatic tenderness and hepatomegaly); thus, this was excluded as a reason for cholestatic hepatic injury.

ALP is the commonest liver enzyme to be increased in hyperthyroid patients at diagnosis (25–64%) [20, 21]. In a study of 30 patients with GD [22], the reported increase in different liver enzymes was as follows: ALP (33%), ALT (26%), GGT (24%), AST (17%), and total bilirubin (8%). The initial increase in ALP may originate from the liver, bone, or both, so it is important to check for GGT and bilirubin levels to diagnose cholestasis [21]. With treatment of hyperthyroidism, improvement of GGT has been reported while the ALP level was increasing, mainly from the bone [20]. The ALP level can take several months to normalize after euthyroidism is reestablished; this is because of increased osteoblast activity [20]. However, our patient's ALP after three months of treatment was mainly of liver origin.

The possible mechanisms of liver injury in hyperthyroidism are as follows [22, 23]: (i) relative hypoxia due to increased oxygen demand while the splanchnic blood supply is unchanged, leading to liver impairment and cholestasis in the centrilobular hepatocytes; (ii) congestive heart failure; (iii) direct toxicity by high thyroid hormones, although this has not been confirmed; and (iv) associated autoimmune liver disease.

Treatment of hyperthyroidism in patients with raised liver enzymes is challenging, as ATDs are hepatotoxic (0.5%) [24]. Carbimazole and methimazole usually cause cholestasis while propylthiouracil usually causes hepatocyte damage by an idiosyncratic mechanism unrelated to the dose [19, 24]. Thus, it is advised to investigate for a concomitant liver disease; if the workup is negative, thionamides can be used with careful monitoring; otherwise it is better to use an alternative, such as radioactive iodine ablation or thyroidectomy [2].

### 3.3. Pulmonary Hypertension

The association between hyperthyroidism and PH was first described in 1950 [25]. PH can be found in patients with GD or nodular goiter with hyperthyroidism [26]. In one study, PH was the most common cardiac complication detected by echocardiography in hyperthyroid patients [27], with a reported prevalence of 36–65% [28, 29]. However, most cases are mild and asymptomatic and are not identified in daily practice [27].

At presentation, the patient had bilateral basal lung crepitations indicative of left ventricular compromise; this can occur with hyperthyroidism. However, the left ventricular dysfunction was mild; the crepitations disappeared on the second day of hospitalization and the ejection fraction was 50–55% on initial echocardiographic examination. On the other hand, the lower limb edema secondary to right-sided heart failure took 3 months to resolve, indicating that this was the main impairment of cardiac function.

Our patient demonstrated an improvement of her RVSP, from 60.16 mmHg at presentation to 52.64 mmHg after 3 months of carbimazole with the resolution of hyperthyroid state. The weight changes that the patient experienced were interesting, as she initially lost weight secondary to hyperthyroidism but then regained some weight because of the right-sided heart failure with resultant edema. Subsequently, once treatment for GD had been initiated, unlike other patients who often gain weight, our patient lost weight as her right heart failure and edema improved. Most cases of PH recover by the time euthyroidism is reestablished. In one report, 79.2% of patients had normal pulmonary artery pressures after 11–21 weeks of ATD treatment [27]. However, in other reports, 3–14 months were needed until normalization of PH occurred [30, 31].

Theories to explain the relationship between hyperthyroidism and PH [32, 33] include the following: (i) autoimmune-mediated endothelial remodeling; (ii) mechanical endothelial damage caused by the high cardiac output; (iii) accelerated metabolism of pulmonary vasodilators (nitric oxide and prostacyclin); (iv) inhibited metabolism of pulmonary vasoconstrictors (endothelin-1, serotonin, and thromboxane); and (v) enhanced pulmonary vascular response to catecholamines. It has been found that pulmonary artery systolic pressure has a significant linear correlation with thyroid-stimulating hormone receptor antibody, pulmonary vascular resistance, and cardiac output [28].

In a study by Marvisi et al. [26] the group of patients who received methimazole showed a more rapid improvement in PH than those who received partial thyroidectomy without pretreatment with methimazole or *β*-blockers; the difference in the pulmonary pressure drop was significant after 15 days, but not after 90 days, of treatment. The methimazole effect may be due to its ability to suppress Ng-nitro-l-arginine methyl ester production, which acutely inhibits nitric oxide synthesis, resulting in increased levels of nitric oxide [34]. In addition, methimazole might cause direct vasodilatation of the pulmonary vasculature [26]. The possibility of thyroid dysfunction should be considered in patients with unexplained PH, as long-standing untreated hyperthyroidism may lead to refractory PH [32, 35].

## 4. Conclusion

Patients with GD may present with rare manifestations that have partially understood mechanisms. With ATDs treatment, pancytopenia should resolve as euthyroidism is reestablished. Liver enzyme levels and PH may normalize, or at least show some improvement, once normal thyroid function is established, with full recovery several months later.

When treating patients with GD, it is generally recommended that liver function tests and a complete blood count be performed before initiating ATDs [13]. Having this baseline information gives insight about the abnormal laboratory findings primarily related to the hyperthyroid state, which is reversible with ATD treatment, and eliminates doubt around whether subsequent abnormal test results are secondary to the use of ATDs.

It is crucial to test the thyroid function of a patient presenting with any of the complications described in our patient where no clear explanation is found. Lack of awareness of such associations with GD may lead to misdiagnosis or delayed diagnosis, with a redundant workup being performed [2].

## Figures and Tables

**Table 1:** Laboratory investigations at presentation and in the 3 months following carbimazole treatment.

	Presentation	Next day	2 weeks	3 months
TSH (mIU/L)	<0.01		<0.01	<0.01
FT4 (pmol/L)	54.69		23.99	6.6
FT3 (pmol/L)	>46.08		12.98	3.97
WBC (×10^9^/L)	2.9	3.6	3.9	6.8
Neutrophils (×10^9^/L)	1.1	2.2	2.1	3.8
Hemoglobin (g/L)	84	96	87	111
Hematocrit (%)	26.6	33.7	30.4	36.7
MCV (femtoliter)	83.1	91.9	92.3	89.1
Platelets (×10^9^/L)	113	143	211	285
T. bilirubin (*µ*mol/L)	35.3	31.1	21.9	11.7
D. bilirubin (*µ*mol/L)	27.4	23.2	17.6	7.3
I. bilirubin (*µ*mol/L)	6.9			
ALP (U/L)	304	262	303	311
GGT (U/L)			128	98
ALT (U/L)	14	13	12	18
AST (U/L)	24	25	16	26
Albumin (g/L)	29	25	25	34
Weight (kg)	96			91

TSH: thyroid-stimulating hormone (0.45–4.5** **mIU/L), FT4: free T4 (9–20** **pmol/L), FT3: free T3 (2.6–5.7** **pmol/L), WBC: white blood cells (4–10 × 10^9^/L), neutrophils (2–7 × 10^9^/L), hemoglobin (120–150** **g/L), hematocrit (36–46%), MCV: mean corpuscular volume (83–101 femtoliter), platelets (150–400 × 10^9^/L), T. bilirubin: total bilirubin (3.4–20.5** ***µ*mol/L), D. bilirubin: direct bilirubin (0–8.6 *µ*mol/L), I. bilirubin: indirect bilirubin (0–3** ***µ*mol/L), ALP: alkaline phosphatase (40–150** **U/L), GGT: gamma-glutamyl transferase (9–36** **U/L), ALT: alanine aminotransferase (0–55** **U/L), AST: aspartate aminotransferase (5–34** **U/L), albumin (35–50** **g/L).
